# Support for Conciliatory Policies in the Israeli-Palestinian Conflict: The Role of Different Modes of Identification and Territorial Ownership Perceptions

**DOI:** 10.3389/fpsyg.2021.769643

**Published:** 2022-01-05

**Authors:** Nora Storz, Borja Martinović, Nimrod Rosler

**Affiliations:** ^1^Department of Interdisciplinary Social Science, ERCOMER, Utrecht University, Utrecht, Netherlands; ^2^Program in Conflict Resolution and Mediation, Tel Aviv University, Tel Aviv, Israel

**Keywords:** ingroup superiority, ingroup attachment, collective psychological ownership, conciliatory policies, territorial conflicts

## Abstract

Understanding people’s attitudes toward conciliatory policies in territorial interethnic conflicts is important for a peaceful conflict resolution. We argue that ingroup identification in combination with the largely understudied territorial ownership perceptions can help us explain attitudes toward conciliatory policies. We consider two different aspects of ingroup identification—attachment to one’s ethnic ingroup as well as ingroup superiority. Furthermore, we suggest that perceptions of ingroup and outgroup ownership of the territory can serve as important mechanisms that link the different forms of ingroup identification with conciliatory policies. In the context of the Israeli-Palestinian conflict, among Israeli Jews (*N* = 1,268), we found that ingroup superiority, but not attachment, was negatively related to conciliatory policies. This relationship was explained by lower outgroup (but not by higher ingroup) ownership perceptions of the territory. Our findings highlight the relevance of studying ingroup superiority as a particularly relevant dimension of identification that represents a barrier to acknowledging outgroup’s territorial ownership, and is thus indirectly related to less support for conciliatory policies in intergroup conflict settings.

## Introduction

With around 100 ongoing disputes over territory (Metrocosm, 2015), territorial conflicts between ethnic groups are rather widespread (Toft, 2014). The relevance of territorial interethnic conflicts became evident again in May 2021 when tensions between Palestinians and Israeli Jews erupted once more in violent clashes. Such conflicts affect the well-being and safety of many (Schmid and Muldoon, 2015), and entities such as policy makers and non-governmental organizations try to find ways to end the conflict. However, conflict resolution might be more realistic if the majority of people living in the regions in question endorse conciliatory policies, such as compromises with the relevant outgroup on concrete policies that will peacefully resolve the conflict.

It has been theorized that certain conflict supporting beliefs, such as outgroup blame for the current situation (Bar-Tal and Halperin, 2011), or the devaluation of the opponent (Ross and Ward, 1995) can stand in the way of support for conciliatory polices. Support for conciliatory policies might also be hindered when individuals strongly identify with their own ethnic group (e.g., Baysu et al., 2018). At the same time, theory and research point at the importance of considering the different ways in which people identify with their group, for instance in a constructive or a destructive way (Staub, 1997). Only the latter form of identification relates to prejudices toward outgroups or outgroup aggression (e.g., Golec de Zavala et al., 2009; Wagner et al., 2012; Verkuyten et al., 2021). Thus, ingroup identification as such might also not represent a barrier for the endorsement of conciliatory policies in conflict regions, but it might be relevant to consider *how* people identify with their own group (see Roccas et al., 2008). Whereas mere attachment to one’s ethnic group need not stand in the way of support for conflict resolution, feelings of ingroup superiority (“our group is better than other groups”) might. We build on previous research on the form of identification in relation to intergroup relations by investigating how ingroup superiority and attachment relate specifically to attitudes toward conciliatory policies.

Further, territorial ownership feelings (“this is our land”) have largely been ignored in the research on intergroup relations in general but also on conflict resolution in particular, yet the question of whom the territory belongs to is central in many interethnic disputes (see ethos of conflict; e.g., Bar-Tal et al., 2012). A handful of recent studies conducted both in peaceful and conflict settings draws attention to the relevance of ownership feelings over one’s country (Brylka et al., 2015; Storz et al., 2020; Nijs et al., 2021) or neighborhood (Torunczyk-Ruiz and Martinović, 2020) for intergroup relations. Additionally, since one needs to feel part of “us” in order to perceive collective ownership, ingroup identification is an important prerequisite for the feeling that something is “ours” (Verkuyten and Martinović, 2017; Storz et al., 2020). However, the role of different dimensions of identification in shaping ownership perceptions has not been considered yet. We propose that ingroup attachment and superiority may relate differently to how people feel about and perceive ownership of the disputed territory which, in turn, could inform agreement with conciliatory policies.

In territorial interethnic conflicts such as the one in Israel/Palestine, perceptions that the territory rightfully belongs to one’s own group tend to be rather prominent (Storz et al., 2020). Yet, we argue that perceiving the ingroup to own a territory does not have to be exclusive. People might at the same time feel that the outgroup has ownership rights to that same territory as well. Ingroup’s perceptions of outgroup’s ownership of the disputed territory in conflict contexts have not been examined yet (but see Storz et al., 2021 for a recent study on shared ownership in Kosovo). Thus, we add to the existing literature on interethnic conflicts by considering not only the role of ingroup, but also of outgroup ownership perceptions in the relation between ingroup superiority and attachment on the one hand, and conciliatory policies on the other hand. We study this in the context of the Israeli-Palestinian conflict, from the perspective of Israeli Jews. In the Israeli-Palestinian conflict, the group that we focus on, Israeli Jews, are the high power group while Palestinians are in a position of having less power in this conflict. For a group with less power, it might be more difficult to acknowledge outgroup ownership and outgroup ownership in addition might have different intergroup outcomes for a group with little power. However, from the perspective of a high power group, a better understanding of the relations between ingroup identification, territorial ownership perceptions, and conciliatory policies can inform theorizing on the role of land ownership perception in support for conflict resolution processes. Further, our findings can help society members of a high power group to tackle territorial conflicts and their resolution.

### Ingroup Identification and Conciliatory Policies

The way people relate to their ingroup can shape intergroup relations. Thus, ingroup identification is a relevant factor to consider when examining popular support for policies aimed at conflict resolution and reconciliation. In the past decades, a nuanced understanding of ingroup identification has been developed that distinguishes between different dimensions of identification and their implications (for a review, see e.g., Ashmore et al., 2004). Specifically, research indicates that individuals can identify with their national group in more constructive (attachment, constructive patriotism) or more destructive (nationalism, blind patriotism, collective narcissism) ways (Staub, 1997; Cichocka, 2016). For instance, the large literature on patriotism and nationalism indicates that, while patriotic individuals are characterized by love and attachment to their nation, nationalistic individuals are characterized by feelings of national superiority (e.g., Kosterman and Feshbach, 1989; Wagner et al., 2012). This line of research has shown that nationalism tends to be related to more prejudicial outgroup feelings, while patriotism is either not related to prejudice, or even to lower levels of prejudice (Wagner et al., 2012). Further, collective narcissism and blind patriotism are related to outgroup aggression and negative attitudes toward minorities and immigrants (Golec de Zavala et al., 2009; Golec de Zavala and Cichocka, 2011; Green et al., 2011; Cichocka, 2016), while national identification and constructive patriotism are unrelated to outgroup aggression (Golec de Zavala et al., 2009), or even positively to outgroup attitudes and tolerance (Verkuyten et al., 2021).

Most of these studies have been conducted in rather peaceful societies. In conflict contexts, and in relation to ethnic groups rather than nations, two modes of identification have been studied in tandem and shown to have contrasting effects on intergroup relations: ingroup superiority and ingroup attachment (Roccas et al., 2006). Ingroup superiority is the feeling that one’s own group is better than other groups (similar to nationalism), whereas ingroup attachment is the affectionate connection to the group (comparable to patriotism). While ingroup superiority is argued to have negative implications for one’s relations with other groups, attachment need not be accompanied by negative intergroup outcomes (Roccas et al., 2008).

Research on the distinction between ingroup attachment and superiority showed that U.S. participants demanded less punishment of an ingroup perpetrator in the Iraqi war and less compensation for the outgroup victim when they felt more ingroup superiority, but stronger attachment to their ingroup was unrelated to demands for punishment and compensation (Leidner et al., 2010). Further, in the context of the Israeli-Palestinian conflict and from the perspective of Israeli Jews, ingroup superiority, but not attachment, was related to higher exonerating cognitions (minimizing ingroup wrongdoings), and to lower perceptions of group-based guilt (Roccas et al., 2006). Acknowledgment of ingroup wrongdoings, as well as group-based guilt were found to be positively related among Israeli Jews to support for conciliatory measures in their conflict with the Palestinians (Čehajić-Clancy et al., 2011; Halperin et al., 2014; Rosler et al., 2018), and thus conciliatory policies might be a next step in conflict resolution processes. Finally, among Serbs, stronger ingroup superiority was related to lower willingness to reconcile with Bosniaks, but ingroup attachment did not relate to reconciliation intentions (Li et al., 2018). Therefore, we expect to find the same pattern of relations when considering conciliatory policies in other contexts of intergroup conflict. We hypothesize that higher ingroup superiority is related to more disagreement with conciliatory policies (H1) and we explore if mere attachment to the ingroup, net of superiority, is related to conciliatory policies.

### Ingroup Identification and Territorial Ownership Perceptions

In addition to group identification, we consider the role of collective psychological ownership of territory—the perception that a specific territory belongs to a certain group (Verkuyten and Martinović, 2017). When one feels part of “us,” the perception that something is “ours” is intuitive, and one does not have to legally own something in order for ingroup ownership perceptions to come about (Rose, 1985; Merrill, 1998; Storz et al., 2020). Further, people do not only perceive something as being “ours” but can also recognize and acknowledge that something belongs to someone else (Kanngiesser and Hood, 2014). Even children understand that it is unacceptable to use someone else’s object (Nancekivell and Friedman, 2017), and they reason for land ownership, also of others (Verkuyten et al., 2015). Thus, children recognize ownership of territory by others. Just as it is possible to acknowledge ownership by another individual, it is possible to acknowledge that an object or a place is owned by another group.

The two modes of identification are expected to be differently related to ingroup and outgroup ownership perceptions of contested territory. Ingroup attachment tends to relate to ingroup favoritism (social identity theory, Tajfel and Turner, 1979). When one is attached to one’s ingroup, one is more focused and concerned about the ingroup, including their norms and values (Brewer, 2001), but also their rights and entitlements (Wenzel, 2000). In territorial conflict regions, the ingroup might be seen as being entitled to own the land. Empirically, ingroup attachment has been found to relate to ingroup ownership perceptions of territory (Brylka et al., 2015; Storz et al., 2020). Thus, we hypothesize that ingroup attachment is related to stronger perceptions of ingroup ownership of the territory (H2). To our knowledge, ingroup attachment has not yet been examined in relation to outgroup ownership perceptions. Individuals who are relatively strongly attached to their ingroup tend to have a strong and positive ingroup orientation but do not have to be negative toward outgroups (Brewer, 2001; Livingstone and Haslam, 2008; Golec de Zavala et al., 2009). Ingroup attachment is ingroup focused and might be relatively independent of outgroup perceptions. However, in intergroup conflict situations stronger ingroup attachment might go together with more negative outgroup perceptions including the rejection of outgroup entitlements (Brewer, 2001; Baysu et al., 2018). Thus, we will explore the relation between ingroup attachment and outgroup ownership perceptions.

The perception that the own group is superior to other groups goes beyond having a sense of belonging to one’s ingroup. Ingroup superiority entails a comparison of the own group with other groups (we are better than others) and is accompanied by judging other groups in light of one’s own group (Roccas et al., 2006). Perceiving one’s ingroup to be superior to others can also be used as a justification for power differences and differences in rights and entitlements between groups (Hagendoorn, 1993). It means that one has the tendency to uncritically glorify one’s ingroup, which may, in turn, relate to legitimization of ingroup rights and entitlements (Roccas et al., 2006) at the expense of the outgroup. Thus, ingroup superiority might inform perceptions of ingroup as well as outgroup rights and entitlements. Consequently, we hypothesize that ingroup superiority is simultaneously related to stronger ingroup (H3) and weaker outgroup ownership perceptions (H4).

### Territorial Ownership Perceptions and Conciliatory Policies

Political resolution of territorial conflicts necessarily involves some kind of agreement on the territorial question (Newman, 2006). Depending on who is perceived to own contested territory, concessions might be easier or more difficult to support. Ownership perceptions shape relationships not only between the owners and the target of ownership (in this case, a territory) but also between individuals and groups in relation to the things that are owned (Blumenthal, 2010). Group ownership entails certain rights and entitlements, such as the right to occupy or use what is perceived to be owned, but also, for instance, to prevent others from using it (Snare, 1972; Merrill, 1998). Thus, the perception that a particular territory is “ours” is likely to have negative consequences for one’s relations with newcomers or other groups living on that territory (Verkuyten and Martinović, 2017).

In line with this reasoning, ingroup ownership perceptions among native populations in Western European contexts have been found to be associated with more negative attitudes toward immigrants (Brylka et al., 2015; Torunczyk-Ruiz and Martinović, 2020; Nijs et al., 2021). Similarly, in conflict contexts, people who perceive more strongly that the contested territory belongs to their ingroup tend to be less willing to forgive members of the rival outgroup or to promote positive intergroup relations (Storz et al., 2020). Extending this initial research to support for conciliatory policies, we hypothesize that stronger ingroup ownership perceptions are associated with less support for conciliatory polices (H5).

Theoretically, outgroup ownership perceptions should go hand in hand with the perception that the outgroup has the same set of rights that generally accompany ingroup ownership. Thus, when one perceives another group to own a territory, one might also see this group as having the right to use the territory and to decide about what happens with the territory (Snare, 1972; Merrill, 1998). These rights might be granted by supporting conciliatory policies. Research has shown that in settler societies people of White European origin with higher perceptions of indigenous (outgroup) ownership of the land are more willing to offer territorial compensation to indigenous people (Nooitgedagt et al., 2021), thereby extending these groups’ land rights. Similarly, there is support for the idea that outgroup ownership perceptions relate to conciliatory policies among Israeli Jews. Higher perception of outgroup (Palestinian) ownership of the West Bank—assessed indirectly as “acknowledging occupation of the territory” by the ingroup (Israeli Jews)—was associated with more support for a compromise in the Israeli-Palestinian conflict, such as return to the 1967 borders and division of Jerusalem (Rosler et al., 2018). Thus, we hypothesize that stronger outgroup ownership perceptions are associated with more support for conciliatory policies (H6).

Bringing all arguments together, the negative relationship between ingroup superiority and support for conciliatory policies is expected to be explained by higher ingroup and lower outgroup ownership perceptions. Further, we expect ingroup attachment to be positively related to ingroup ownership perceptions, and via higher ingroup ownership indirectly to less support for conciliatory policies, and we explore the relation between attachment and outgroup ownership perceptions.

### The Conflict Context

We tested our hypotheses among Israeli Jews, in the context of one of the most protracted interethnic conflicts in the world—the Israeli-Palestinian conflict. The conflict originated when the Jewish national movement known as Zionism emerged in the nineteenth century, aiming at the establishment of a Jewish state in Palestine/Land of Israel based on the ancient bonds of Jews to the land (Rouhana and Bar-Tal, 1998; Waxman, 2019). While at that time Jews were a small minority in Palestine, the increasing Zionist remigration to the land with concrete national-political goals escalated the conflict between the communities. It erupted in a war in 1947, after the United Nations declared the partition of the land into a Jewish and an Arab state. The partition was endorsed by the Jewish community who proclaimed its independent state in 1948, and rejected by the Palestinians and Arab countries (Tessler, 2009). In 1949, the territory of the State of Israel was determined along armistice lines, which have since been disputed, and increasingly so after Israel’s occupation of the West Bank and the Gaza Strip during the 1967 war (Rosler et al., 2018). Currently, the land dispute is mainly about the question where the borders of Israel should be, rather than about the question of existence of Israel (Waxman, 2019). Therefore, looking at Israeli Jew’s perceptions of both Jewish and Palestinian ownership of the land between the Jordan River and the Mediterranean Sea is relevant to better understand Israeli Jews’ attitudes toward conflict resolution.

## Materials and Methods

### Data and Participants

Data were collected among Jewish Israelis by the research agency Kantar in collaboration with a local partner agency, Profiles, in October 2020. Profiles administers a panel of participants who regularly participate in their research. They sent out emails to Jewish panel members who were 18 years and older. Additionally, they posted the survey on the Profiles website, and panel members who did not receive a direct invitation but were eligible could access and fill out the questionnaire in this way.^1^ Participants completed an online questionnaire in Hebrew using the method of computer-assisted web interviewing (CAWI) and in exchange for a modest compensation in line with the agency’s usual practices.

Our sample consists of 1,268 Jewish Israelis.^2^ In terms of gender and age (ranging from 18 until 87 years) the sample resembles the Israeli Jewish population (see Central Bureau of Statistics, 2020). The sample was also diverse in terms educational level and degree of religiosity (Table 1). Moreover, participants from different regions were targeted.^3^ The research agency provided weights based on participants’ age, gender, and their region of residence, and all results presented below were obtained using these weighted data.

**TABLE 1:** Demographic composition of the sample in terms of gender, age, educational level and religiosity.

Indicator	
**Male (vs. female)**	**50%**
**Age**	**44.65 (16.16)**
**Educational level**	
Primary	10%
Secondary	41%
Tertiary	49%
**Religiosity**	
Secular	55%
Traditional—not so religious	19%
Traditional religious	7%
Religious	13%
Ultra-orthodox	6%

*N = 1 participant indicated another gender, recoded here as missing. N = 44 participants had missing values on educational level, and N = 11 participants had missing values on religiosity.*

### Measures

*Ingroup attachment* was assessed by three items inspired by Roccas et al. (2006): “My Jewish origin is an important part of me,” “Belonging to the Jewish people defines who I am” and “I strongly identify with other people with Jewish ancestry.” Answers have been provided on 7-point scales (1 = “Strongly disagree” to 7 = “Strongly agree”) and the items formed a reliable scale (ρ = 0.89; Raykov, 2004).

*Ingroup superiority* was also assessed by three items, following Roccas et al. (2006): “Jews are better than other groups in many respects,” “Other groups can learn a lot from Jews,” and “Jews are the chosen people” (ρ = 0.87; 1 = “Strongly disagree” to 7 = “Strongly agree”).

*Ingroup* and *outgroup ownership perceptions* were assessed by three items each, drawing on Storz et al. (2020). Following an introduction (“Both Israeli Jews and Palestinians live in the land from the Jordan River to the Mediterranean Sea and there is disagreement in how far the land belongs to either group. We would like to hear your opinion on this.”), participants were asked “How much does this land belong to the following groups?” “To what extent can each of the following groups be seen as the rightful owner of this land?” and “How much can each of these groups claim this land for themselves?.” Each item was asked for Jewish (ingroup) and Palestinian (outgroup) ownership (1 = “Not at all” to 7 = “Very much”). Ingroup as well as outgroup ownership perceptions formed reliable scales (ρ = 0.87 and 0.91, respectively).

*Support for conciliatory policies* was assessed by three items, drawing on Yaar and Rosler (2019). Participants were asked in how far they oppose or support “The creation of an independent Palestinian state alongside the State of Israel.” Answers were given on 7-point scale (1 = “Strongly oppose” to 7 = “Strongly support”). Further, they indicated to what extent they were in favor or against “negotiations for peace between Israel and the Palestinian Authority” and convinced or not convinced “that negotiations between Israel and the Palestinian Authority will lead to peace between Israel and the Palestinians in the coming years?” Answers were given on 4-point scales (1 = “Very much in favor” to 4 = “Very much against”; and 1 = “Very much convinced” to 4 = ‘‘Not convinced at all’’). We re-scaled these two items into 7-point scales to match the first item.^4^ The factor assessing support for conciliatory policies had a good reliability (ρ = 0.76).

We included several control variables. First, we controlled for substantive variables which have been found to relate to one or more of the outcome variables in our model. *Place attachment* has been shown to be related to ingroup ownership perceptions (Storz et al., 2020) and to acceptance of outgroups (Torunczyk-Ruiz and Martinović, 2020). We assessed place attachment with three items: “The land from the Jordan River to the Mediterranean Sea feels like my home,” “I feel attached to this land,” and “I would regret having to leave this land” (ρ = 0.82; Lewicka, 2010; Torunczyk-Ruiz and Martinović, 2020). Moreover, since attitudes toward political compromise have been shown to be influenced by *political orientation* among Israeli Jews (Hermann and Yuchtman-Yaar, 2002), we controlled for a left-right scale of political orientation (1 = “left-wing,” 2 = “moderate left-wing,” 3 = “center,” 4 = “moderate right-wing,” 5 = “right-wing”). Furthermore, the rivaling groups in the Israeli-Palestinian conflict belong to different religious groups (Judaism vs. Islam), and previous research in this context has shown that stronger religiosity is related to lower agreement with compromise (Maoz and Eidelson, 2007). Therefore, we controlled for participants’ *degree of religiosity*. We contrasted secular participants (reference category; the largest group among Jewish Israelis) with traditional—not so religious, traditional religious, religious, and ultra-orthodox participants. Second, we controlled for the standard demographic variables *age* (continuous), *gender* (1 = male, 0 = female), and *educational level* (primary, secondary, and tertiary, with secondary as reference category).^5^ Finally, half of the sample also took part in an (unsuccessful) experiment that focused on a different research question than the one addressed here. More details on the experiment can be found in Supplementary Material 1. We controlled for 1 = experimental condition and 0 = baseline. The latter contains participants who were in the control condition of the experiment, as well as participants who received the cross-sectional version of the survey.

## Results

### Measurement Model

We used latent variables for all multi-item constructs, including the control variable place attachment. We conducted a confirmatory factor analysis (CFA) in M*plus* version 8.0 using weighted data, to assess whether all variables loaded on the intended factors. A model with six distinct latent factors—ingroup attachment, ingroup superiority, ingroup ownership perceptions, outgroup ownership perceptions, support for conciliatory policies, and place attachment—did not fit the data very well [χ^2^(df) = 765.22 (120), *p* < 0.001; RMSEA (90% CI) = 0.065 (0.068,0.079); CFI = 0.930; SRMR = 0.040]. The modification indices suggested to free the covariance between the third items assessing ingroup and outgroup ownership perceptions. Since this is the only item of the scale that might be interpreted as rather exclusive ownership, we deemed this step justifiable. A model where the covariance was freed fitted the data adequately [χ^2^(df) = 579.99 (119), *p* < 0.001; RMSEA (90% CI) = 0.055 (0.051,0.060); CFI = 0.950; SRMR = 0.040], and significantly better than the model where the covariance was not freed [Δχ^2^(df) = 98.81 (1), *p* < 0.001] (Putnick and Bornstein, 2016). In this final model, all standardized loadings were 0.57 or higher, and the respective factors explain at least 33% of the variance in each item.^6^

### Descriptive Findings

Israeli Jews were on average attached to their ingroup and felt that their ingroup was superior to other groups (see Table 2). Moreover, they perceived on average that the contested territory belonged to their ingroup, but not to Palestinians. Ingroup ownership perceptions were significantly stronger than outgroup ownership perceptions [pairwise comparison, Wald (1) = 3681.92, *p* < 0.001]. Participants on average slightly, but significantly, disagreed with conciliatory policies.

**TABLE 2:** Means, standard deviations, and Wald tests^a^ of the main constructs and theoretically relevant control variables^b^ and their correlations (*N* = 1,268).

	*M*	*SD*	*Wald test*	1.^c^	2.	3.	4.	5.	6.
1. Ingroup attachment	5.86	1.28	2641.70 (1)***	–					
2. Ingroup superiority	4.55	1.74	127.25 (1)***	0.75*** (0.72, 0.79)	–				
3. Ingroup ownership perceptions	6.28	1.10	5373.33 (1)***	0.56*** (0.51, 0.64)	0.53*** (0.48, 0.58)	–			
4. Outgroup ownership perceptions	2.59	1.53	1098.11 (1)***	−0.43*** (−0.49, −0.37)	−0.54*** (−0.59, −0.49)	−0.37*** (−0.43, −0.31)	–		
5. Support for conciliatory policies	3.85	1.50	12.79 (1)***	−0.44*** (−0.50, −0.39)	−0.64*** (−0.69, −0.59)	−0.39*** (−0.45, −0.33)	0.61*** (0.55, 0.66)	–	
6. Place attachment	5.95	1.18	3545.15 (1)***	0.64*** (0.58, 0.70)	0.52*** (0.46, 0.57)	0.56*** (0.49, 0.62)	−0.33*** (−0.39, −0.27)	−0.28*** (−0.34, −0.21)	−
7. Political orientation	3.59	1.09	351.65 (1)***	0.50*** (0.45, 0.55)	0.64*** (0.60, 0.68)	0.43*** (0.38, 0.48)	−0.54*** (−0.59, −0.50)	−0.68*** (−0.72, −0.64)	0.36*** (0.31, 0.42)

***p < 0.001. Political orientation had N = 43 missing values. Political orientation was assessed on a 5-point scale (1 = left-wing to 5 = right-wing). All other constructs are on 7-point scales, with lower values indicating less agreement.

*^a^We used the Wald test to assess significant differences between the mean and the scale midpoint of each construct.*

*^b^Only continuous control variables are displayed. For degree of religiosity (see Table 1).*

*^c^95% confidence intervals for the correlations are presented in brackets.*

Ingroup attachment and superiority were strongly positively correlated (see Table 2). Further, the bivariate correlations between ingroup attachment and ingroup ownership perceptions were positive, while ingroup attachment was negatively correlated with outgroup ownership perceptions as well as with conciliatory policies. The same pattern was observed for ingroup superiority. Ingroup and outgroup ownership perceptions were negatively correlated with each other. Further, ingroup ownership was negatively correlated with support for conciliatory policies, while outgroup ownership was positively correlated. See Table 2 also for correlations with continuous control variables place attachment and political orientation.

### Structural Model

**FIGURE 1:**
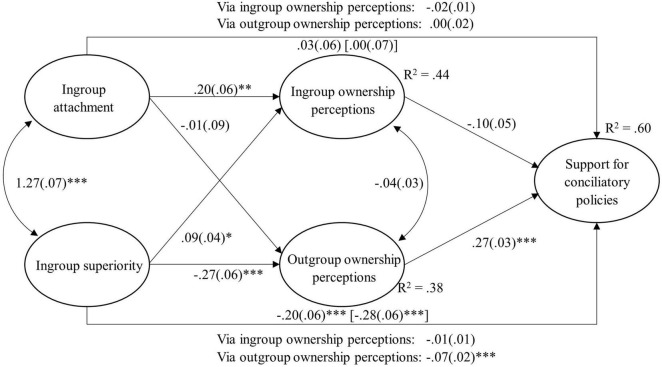
Unstandardized results of a structural equation model explaining support for conciliatory policies (*N* = 1,268). Total effect shown in square brackets; *R*^2^ is the explained variance of the latent outcome variables; *p < 0.05, ^**^p < 0.01, ^***^p < 0.001. Model fit: [χ^2^(df) = 1090.46 (261)^***^, CFI = 0.935, SRMR = 0.042, RMSEA (90% CI) = 0.050, (0.047, 0.053)]. For results of the control variables (see Table 3).

We used the weighted data and analyzed them in M*plus* version 8.0, using the Robust Maximum Likelihood estimator (MLR; Muthén and Muthén, 1998–2012) to account for the skewness in ingroup and outgroup ownership perceptions (Lei and Shiverdecker, 2020), and using full maximum likelihood estimator (FIML) to account for missing values in the manifest variables political orientation, degree of religiosity, and educational level (see note underneath Tables 1, 2). We used latent variables and estimated a mediation model to test our hypotheses. We specified paths from ingroup attachment and ingroup superiority to ingroup and outgroup ownership perceptions as well as to support for conciliatory policies. Further, we specified a path from ingroup ownership and a path from outgroup ownership to conciliatory policies. Results of this structural model can be found in Figure 1, where we show the hypothesized paths only. For the results of the control variables (please see Table 3).

**TABLE 3:** Unstandardized results of the control variables of the structural equation analysis explaining support for conciliatory policies (*N* = 1,268).

	Ingroup	Outgroup	Support for
	ownership	ownership	conciliatory
	perceptions	perceptions	policies
	
	*b (SE)*	*b (SE)*	*b (SE)*
Place attachment	0.25 (0.05)***	−0.07 (0.06)	0.16 (0.05)**
Political orientation	0.11 (0.03)***	−0.44 (0.05)***	−0.42 (0.05)***
**Degree of religiosity (Ref. secular)**			
Traditional (Not so religious)	−0.03 (0.05)	0.06 (0.10)	−0.08 (0.10)
Traditional religious	−0.12 (0.09)	0.38 (0.14)**	0.06 (0.15)
Religious	−0.09 (0.06)	0.01 (0.13)	−0.58 (0.14)***
Ultra-orthodox	−0.23 (0.09)*	−0.09 (0.14)	−0.44 (0.19)*
Male (vs. female)	−0.03 (0.04)	0.00 (0.07)	−0.05 (0.07)
Age	0.00 (0.00)	−0.004 (0.002)*	0.01 (0.00)***
**Education (Ref. secondary)**			
Primary	0.21 (0.07)**	−0.06 (0.13)	0.00 (0.13)
Tertiary	−0.08 (0.05)	0.09 (0.08)	−0.07 (0.08)
Experimental condition (vs. baseline)	0.17 (0.04)***	0.15 (0.07)*	−0.02 (0.07)

*p < 0.05, **< 0.01, ***p < 0.001. For results of the main variables (see Figure 1).

As expected, ingroup superiority had a total negative relation with support for conciliatory policies (H1), while ingroup attachment was unrelated to conciliatory policies. Furthermore, stronger attachment to Israeli Jews was related to more ingroup ownership perceptions (H2), but not to weaker outgroup ownership perceptions. However, stronger agreement with ingroup superiority was related to stronger ingroup (H3) and weaker outgroup ownership perceptions (H4). Stronger ingroup ownership perceptions were descriptively related to lower support for conciliatory policies but just below the significance threshold (*p* = 0.051; H5). Stronger outgroup ownership perceptions were significantly related to more support for conciliatory policies (H6). Overall, we detected a negative direct effect of ingroup superiority on conciliatory policies as well as a negative indirect effect via outgroup ownership perceptions (see Figure 1). None of the other indirect effects were significant. Thus, we conclude that those who felt that Jews were superior to other groups supported conciliatory policies less because they agreed less that Palestinians own the land between the Jordan River and the Mediterranean Sea.

Regarding substantial control variables, participants who were more attached to the land between the Jordan River and the Mediterranean Sea perceived more that the territory belongs to Jews, but they were also more in favor of conciliatory policies (see Table 3). The more right-wing oriented participants were, the more ingroup and the less outgroup ownership they perceived, and the less they were in favor of conciliatory policies. Participants’ degree of religiosity was less conclusively related to the three endogenous variables. As compared to secular participants, traditional religious participants had higher outgroup ownership perceptions, ultra-orthodox participants had lower ingroup ownership perceptions,^7^ and religious and ultra-orthodox participants agreed less with conciliatory policies. For results of the demographic control variables (please see Table 3).

Additionally, we re-estimated the model without the control variables. Results can be found in Supplementary Figure 1. We only report differences in results as compared to the model depicted in Figure 1. The direct relation between ingroup attachment and agreement with conciliatory policies was positive and significant (*b* = 0.17, *SE* = 0.06, *p* = 0.005) as compared to positive but small and non-significant in the original model. Thus, in this model without accounting for control variables, the pattern of the findings remained the same, but we detected one result that changed in terms of the level of significance.

## Discussion

In the context of the Israeli-Palestinian conflict, we examined whether Israeli Jews’ territorial ownership perceptions of the land between the Jordan River and the Mediterranean Sea explain the relation between ingroup identification and support for conciliatory policies. With this study we added to the literature on conflict resolution and reconciliation, and intergroup relations in a broader sense, in two ways. First, we distinguished between ingroup attachment and ingroup superiority as two dimensions of identification that were expected to differently relate to conciliatory policies. While previously, these two dimensions of identification have been researched in relation to outgroup prejudice or aggression and to group-based guilt (Roccas et al., 2006, 2008; Golec de Zavala et al., 2009; Wagner et al., 2012), we add to the literature by relating them to agreement with conciliatory policies in a conflict setting. Second, we zoomed in on the explanatory role of ingroup and outgroup territorial ownership perceptions. Research on these ownership perceptions is still in its infancy. One study has addressed the role of ingroup ownership perceptions in reconciliation intentions (Storz et al., 2020), and one other study has addressed the role of outgroup ownership perceptions only and did so using proxy measures (Rosler et al., 2018). Ours it the first study to consider both of these perceptions in tandem. We found that Israeli Jews who felt more strongly that their group was superior to other groups were less willing to support conciliatory policies, while those who were more attached to their group did not differ in their support for conciliatory policies from those who were less attached. When control variables were not included in the model, ingroup attachment was even related to more support for conciliatory policies. These findings resonate with our theoretical reasoning that it is not identification with one’s group as such, but rather the element of positive accentuation of the own group against others that is detrimental to positive intergroup relations (Roccas et al., 2008; Golec de Zavala et al., 2009; Wagner et al., 2012; Cichocka, 2016). Attachment to one’s ethnic group, when feelings of ingroup superiority are being accounted for, might even provide secure grounds for openness to another group’s needs (Golec de Zavala et al., 2013; Verkuyten et al., 2021).

The relevance of distinguishing between ingroup superiority and attachment was further emphasized when we considered how these two dimensions related to ingroup and outgroup ownership perceptions. We found that Israeli Jews who more strongly perceived that their group was superior to other groups regarded the contested land from the Jordan River to the Mediterranean Sea to belong more to their ingroup and less to Palestinians. However, when participants were more attached to their ingroup, in line with theory (Brewer, 2001) and research (Storz et al., 2020), they perceived that the land more strongly belonged to Jews, but they did not differ from those who were less attached in their perceptions of Palestinian ownership of that land. These findings resonate with our theoretical expectations that, when one perceives one’s own group to be superior, one regards the ingroup as more, and the relevant outgroup as less deserving (Kosterman and Feshbach, 1989; Roccas et al., 2006; Leidner et al., 2010). Overall, our findings that contrast ingroup attachment and ingroup superiority are in line with recent theories and research on different modes of identification, supporting the idea that attachment to one’s group (similar to findings on patriotism) is not related to negative outgroup consequences, while superiority perceptions of one’s own group (similar to nationalism and collective narcissism) are (Roccas et al., 2008; Golec de Zavala et al., 2009; Wagner et al., 2012; Cichocka, 2016).

Furthermore, we showed that Israeli Jews who acknowledged Palestinian ownership of the contested territory more were also more in favor of policies aimed at resolving the territorial conflict (Rosler et al., 2018). At the same time, perceiving more strongly that the Jewish ingroup owns the land was negatively related to these policies (Verkuyten and Martinović, 2017; Storz et al., 2020) but just not significantly so. Acknowledgment of outgroup ownership was possibly a more relevant explanatory mechanism than ingroup ownership because the latter was quite strongly perceived in our sample, whereas we found more variation in outgroup ownership perceptions. Especially in territorial conflict regions, ingroup ownership of the territory might be considered a given, making outgroup ownership perceptions more relevant to consider when researching conciliatory attitudes.

### Limitations and Future Directions

There are several limitations of our study that could be addressed in future research. First of all, we used cross-sectional data, which does not allow us to draw conclusions regarding the causality between the variables considered. We have theoretical reasons to believe that ingroup superiority predicts ownership perceptions, which in turn predict agreement with conciliatory policies (Roccas et al., 2008; Pierce and Jussila, 2010; Verkuyten and Martinović, 2017), and other cross-sectional mediation studies have examined the same causal order and found significant paths from identification to ownership (Martinović and Verkuyten, 2013; Brylka et al., 2015; Li et al., 2018). Further, there is first experimental evidence showing that shared ownership perceptions (“this land belongs to both groups”) increase reconciliation intentions (Storz et al., 2021). Nevertheless, reverse directions of influence are possible. Ownership claims could for instance also be used as a justification for opposition to conciliatory policies, and the identification with one’s ingroup could increase when what is perceived to be “ours” is threatened, as it is in territorial conflicts. Since reverse mediation testing is not statistically meaningful or informative when it comes to drawing conclusions about causality (Thoemmes, 2015; Lemmer and Gollwitzer, 2017; Rohrer et al., 2021), experimental and longitudinal research is needed.

Relatedly, our findings only support the mediation model to a limited extent, with only the path from ingroup superiority to support for conciliatory policies being mediated by outgroup ownership perceptions. While we did not have clear expectations regarding the relation of ingroup attachment with conciliatory policies via outgroup ownership perceptions (Roccas et al., 2008; Golec de Zavala et al., 2009), we also did not find a mediation via ingroup ownership perceptions. Further, and unexpectedly so, we did not find a mediation of ingroup superiority to conciliatory policies via ingroup ownership perceptions either. These findings do not only call for experimental and longitudinal research for more conclusive results regarding the causal relationship between the different constructs, but also for more research on possible antecedents of ownership perceptions in conflict contexts. For instance, ownership principles could predict ingroup and outgroup ownership perceptions, and could in turn relate to conciliatory policies. Ownership principles are reasons that can be used to argue for group ownership, such as the principle of having been first or having invested into the land (Verkuyten and Martinović, 2017). While having been first is a rather exclusive ownership argument—only one group can have been the first—having invested into the land can be more inclusive—two groups could have invested together—and could thus relate to stronger perceptions that a relevant outgroup also owns the territory (Nooitgedagt et al., 2021).

Next, we focused on the perspective of Israeli Jews, whereas ideally we would have also included the Palestinian perspective. To our knowledge, no research is yet available that takes into account both parties involved in a territorial conflict for understanding the role of territorial ownership perceptions for conciliatory attitudes. While we would overall expect our model to work similarly among both parties, there is also a reason to expect possible differences: Palestinians are in a less powerful position than Israeli Jews. Previous research that took a two-sided perspective on conflict resolution processes in the Israeli-Palestinian conflict found significant differences between the two groups that seem to reflect status differences. For instance, Palestinians have been recently shown to be more supportive of militant policies than Israeli Jews, as well as having lower levels of trust toward the outgroup, likely reflecting their inferior position (Shikaki et al., 2020). Additionally, it has been found that active place attachment to Jerusalem was related to more positive attitudes to the outgroup only among Israeli Jews but not among Palestinians. Jews, the high-power group, might be more open to share the space with the low-power group, perceiving to be still in control of the place (Wnuk and Oleksy, 2021). This reasoning could hold for the concept of ownership perceptions as well: it might be more difficult for low-power group members to acknowledge outgroup ownership, and if they do, the question is still whether it would have the same positive effects for intergroup relations. Therefore, it is important for future research to analyze these same relationships while comparing high- and low-power groups.

Finally, while group ownership seems to be a relevant concept to study in the context of territorial conflicts, an empirical issue we encountered is that, not surprisingly, participants agreed with ingroup ownership perceptions rather strongly, while they mostly disagreed with outgroup ownership. In order to further understand the role of ownership perceptions in shaping intergroup relations, experimental research and research in contexts where group ownership is less prominent in people’s minds could be helpful. For example, in regions where there is more variation in ingroup ownership perceptions, like in Western European countries, ingroup ownership is a strong predictor of negative intergroup relations (Nijs et al., 2021). It would be valuable to find ways to further research ownership perceptions in conflict contexts, for instance, by considering perceptions of shared ownership of contested land (see Storz et al., 2021, for first evidence).

## Conclusion

To conclude, with the present research in the context of the Israeli-Palestinian conflict from the perspective of Israeli Jews, we showed that a sense of ingroup superiority (rather than ingroup attachment) can form a barrier to support for conciliatory policies, and thus joins other socio-psychological factors that inhibit its peaceful resolution (see Bar-Tal and Halperin, 2011). This is because people who think their group is better than other groups are less likely to recognize the outgroup (Palestinians) as being entitled to the contested land. Thus, we provide evidence for the pivotal role of ingroup superiority and outgroup ownership perceptions when it comes to understanding support for conciliatory policies better. These findings can provide policy makers, non-governmental organizations, teachers and other relevant actors in territorial conflict regions with important information on how to improve intergroup relations. While there is no need to de-emphasize belongingness and attachment to the ethnic ingroup, it is crucial to communicate to the public that one’s ingroup is not better or worth more than other groups. A reduced feeling of ingroup superiority should relate to more support for conciliatory policies, particularly via stronger outgroup ownership perceptions. Additionally, opening up discussions and debates about ownership rights of the conflicting outgroup might be key to more support for peaceful resolution of territorial conflicts.

## Data Availability Statement

The datasets presented in this study are available at the following link: https://osf.io/neq29/?view_only=ac3a9c1e194c46b498d2d12f1ded7aa1.

## Ethics Statement

The studies involving human participants were reviewed and approved by Utrecht University, Ethics Review Board of the Faculty of Social and Behavioral Sciences, and by Tel Aviv University, The Ethics Committee of the Faculty of Social Sciences. The participants provided their written informed consent to participate in this study.

## Author Contributions

NS: conceptualization, data curation, formal analysis, investigation, methodology, and writing—original draft. BM: conceptualization, funding acquisition, supervision, and writing—review and editing. NR: conceptualization, data collection—translation, and writing—review and editing. All authors contributed to the article and approved the submitted version.

## Conflict of Interest

The authors declare that the research was conducted in the absence of any commercial or financial relationships that could be construed as a potential conflict of interest.

## Publisher’s Note

All claims expressed in this article are solely those of the authors and do not necessarily represent those of their affiliated organizations, or those of the publisher, the editors and the reviewers. Any product that may be evaluated in this article, or claim that may be made by its manufacturer, is not guaranteed or endorsed by the publisher.

## Footnotes

1 As there is no one-on-one relationship between the direct invitations to participate in the surveys administered by Profiles and the number of the panel members ultimately exposed to each survey, Profiles cannot calculate the response rates.

2 Data were collected as part of a larger survey, which is why the final number of participants is larger than needed in order to detect effects for our model. The minimum effect size to detect effects when using a structural equation model with 5 latent variables and an anticipated effect size of 0.2, following Soper’s *a priori* calculation, was *N* = 376. Our larger sample size gave us room to also include some control variables into our model.

3 Participants came from (1) Jerusalem (14%), (2) Central/Gush Dan; including Tel Aviv (25%), (3) North; including Haifa region (21%), (4) Shfela and South (22%), and (5) Sharon (15%). Consequently, our participants are spread out within the official borders of Israel, but do not live in the occupied territories. For 3% of participants, we do not have information about their residence.

4 The formula for re-scaling the items was the following: rescaled_ item = (original_item − 1)*(6/3) + 1.

5 This study was part of a larger survey in which also concepts such as autochthony, religious identification, collective efficacy, intergroup relations [e.g., feelings toward outgroup, intergroup tolerance, intergroup relations between Israelis and Palestinians, emotions, as well as several other items regarding possible solutions to the conflict (e.g., annexation of the Palestinian territories)] have been assessed.

6 Because we are considering two dimensions of ingroup identification—attachment and superiority—we wanted to ensure that we are not dealing with multicollinearity. Therefore, we tested for the variance inflation factor (VIF) in SPSS version 24. The VIF of ingroup attachment was 2.01 and the VIF of ingroup superiority was 2.04, thus both way below the commonly used cut-off value of 10.

7 Ultra-orthodox Jews might perceive less ingroup ownership because they have a stronger connection to the laws of the Torah rather than to the land, or because they relate the ownership of the land to the state of Israel from which many ultra-orthodox Jews feel disconnected.
