# Supplementary material for: Support for Conciliatory Policies in the Israeli-Palestinian Conflict: The Role of Different Modes of Identification and Territorial Ownership Perceptions
Source: Front Psychol. 2022 Jan 5;12:769643. doi: 10.3389/fpsyg.2021.769643 (PMC8766312; doi:10.3389/fpsyg.2021.769643)
Supplement: Supplementary file 1 [file Data_Sheet_1.docx]

**Online supplement**

Online Supplement 1

Half of the participants (*N* = 659) took part in an experiment. The aim of the experiment was to elicit stronger feelings of shared ownership in one group by presenting participants (*N* = 210) with a shared ownership narrative. This was contrasted by a group of people who were presented with an ingroup ownership narrative (*N* = 228). A third group of people was treated as a control group, they did not read any narrative (*N* = 221). However, this experiment did not work as intended, people in all three groups perceived similar levels of shared ownership of the land between the Jordan River and the Mediterranean Sea (F(656, 2) = .689, *p* = .502). Therefore we decided to use the whole sample in the current analyses.

To gain more confidence in doing so, we checked for mean differences in ingroup and outgroup ownership perceptions between these three experimental conditions. While the three groups did not differ in outgroup ownership perceptions (F(656. 2) = 1.368, *p* = .255), they did differ significantly in ingroup ownership perceptions (F(656, 2) = 5.115, *p* = .006). The Tukey HSD post hoc test revealed that participants in the shared ownership condition (M = 6.37, *SD* = .96) as well as those in the ingroup ownership condition (*M* = 6.41, *SD* = .93) reported significantly higher ingroup ownership perceptions than participants in the control condition (*M* = 6.11, *SD* = 1.25); *p* = .037, 95% CI [.01, .49]; *p* = .008, 95% CI [.06, .53]. However, those in the shared ownership condition did not significantly differ from those in the ingroup ownership condition in their ingroup ownership perceptions (*p* = .888, 95% CI [-.29, .19]). Interestingly, in both experimental conditions participants perceived significantly more ingroup ownership than in the control condition. It could be that the issue of land ownership has been made salient in the participants’ minds when they read about either ingroup or shared ownership. To take these findings into account, we controlled for experimental condition (shared ownership and ingroup ownership condition) vs. baseline condition (control condition and cross-sectional data)^[[1]](#footnote-1)^. A t-test revealed that participants in an experimental condition scored significantly higher on ingroup ownership perceptions (*M* = 6.39, *SD* = .944) than participants in the baseline condition (*M* = 6.21, *SD* = 1.17), *t*(1266) = 2.758, *p* = .006. Participants in an experimental condition did not differ significantly (*t*(1266) = 1.727, *p* = .084) in their average perceptions of outgroup ownership (*M* = 2.68, *SD* = 1.55) from participants in the baseline condition (*M* = 2.53, *SD* = 1.44).

Ingroup attachment

Ingroup superiority

Ingroup ownership perceptions

Outgroup ownership perceptions

Support for conciliatory policies

.33(.06)***

-.08(.08)

-.43(.05)***

.12(.03)***

1.28(.07)***

-.09(.03)**

-.10(.05)

.36(.04)****

.17(.06)** [.11(.06)]

-.44(.05)*** [-.61(.02)***]

Via ingroup ownership perceptions: -.03(.02)

Via outgroup ownership perceptions: -.03(.03)

Via ingroup ownership perceptions: -.01(.01)

Via outgroup ownership perceptions: -.16(.02)***

R^2^ = .36

R^2^ = .29

R^2^ = .51

*Note :* Total effect shown in square brackets; R^2^ is the explained variance of the latent outcome variables;

* *p* < .05, ** *p* < .01, *** *p* < .001. Model fit: χ^2^(df) = 472.09(91)***, CFI = .958, SRMR = .035, RMSEA [90% CI] = .054, [.049, .059].

[.043, .049].

Figure S1. Unstandardized results of a structural equation model explaining support for conciliatory policies, without control variables included (*N* = 1268)

1. There was one difference between the control condition in the experimental data on the one hand, and the cross-sectional data on the other hand: ingroup and outgroup ownership were asked at different parts of the questionnaire. Participants in the control condition of the experiment have been asked at the end of the questionnaire (before the demographic questions) in how far they agree or disagree with ingroup and outgroup ownership, rather than in the middle of the questionnaire like participants in the cross-sectional data. Importantly, the pattern of findings for the current paper is the same regardless of whether we include or exclude this control condition in the analyses. [↑](#footnote-ref-1)
